# KDNA Genetic Signatures Obtained by LSSP-PCR Analysis of *Leishmania (Leishmania) infantum* Isolated from the New and the Old World

**DOI:** 10.1371/journal.pone.0043363

**Published:** 2012-08-17

**Authors:** Janaína Sousa Campos Alvarenga, Carla Maia Ligeiro, Célia Maria Ferreira Gontijo, Sofia Cortes, Lenea Campino, Annamaria Ravara Vago, Maria Norma Melo

**Affiliations:** 1 Departamento de Parasitologia, Instituto de Ciências Biológicas, Universidade Federal de Minas Gerais, Belo Horizonte, Minas Gerais, Brasil; 2 Departamento de Morfologia, Instituto de Ciências Biológicas, Universidade Federal de Minas Gerais, Belo Horizonte, Minas Gerais, Brasil; 3 Centro de Pesquisas René Rachou, Fundação Oswaldo Cruz, Belo Horizonte, Minas Gerais, Brasil; 4 Unidade de Leishmanioses, Instituto de Higiene e Medicina Tropical, Universidade Nova de Lisboa, Lisboa, Portugal; Royal Tropical Institute, The Netherlands

## Abstract

**Background:**

Visceral Leishmaniasis (VL) caused by species from the *Leishmania donovani* complex is the most severe form of the disease, lethal if untreated. VL caused by *Leishmania infantum* is a zoonosis with an increasing number of human cases and millions of dogs infected in the Old and the New World. In this study, *L. infantum* (syn. *L.chagasi*) strains were isolated from human and canine VL cases. The strains were obtained from endemic areas from Brazil and Portugal and their genetic polymorphism was ascertained using the LSSP-PCR (Low-Stringency Single Specific Primer PCR) technique for analyzing the kinetoplastid DNA (kDNA) minicircles hypervariable region.

**Principal Findings:**

KDNA genetic signatures obtained by minicircle LSSP-PCR analysis of forty *L. infantum* strains allowed the grouping of strains in several clades. Furthermore, LSSP-PCR profiles of *L. infantum* subpopulations were closely related to the host origin (human or canine). To our knowledge this is the first study which used this technique to compare genetic polymorphisms among strains of *L. infantum* originated from both the Old and the New World.

**Conclusions:**

LSSP-PCR profiles obtained by analysis of *L. infantum* kDNA hypervariable region of parasites isolated from human cases and infected dogs from Brazil and Portugal exhibited a genetic correlation among isolates originated from the same reservoir, human or canine. However, no association has been detected among the kDNA signatures and the geographical origin of *L. infantum* strains.

## Introduction

Visceral leishmaniasis has been described as a growing zoonosis with an increasing number of new human cases and millions of dogs affected in Europe, Asia, North of Africa and in Latin America. More recently, VL caused by *L. infantum* has been considered as an emergent disease in North America [1]. Untreated VL is usually fatal and infected dogs constitute the main domestic reservoir for human transmission, which play an important role in the transmission to humans. In addition, VL can affect immunocomprimised individuals as an opportunistic infection, and consequently coinfections have been reported in several areas of zoonotic transmission [2]. Recent studies suggest that the parasites which cause the disease in different foci with zoonotic transmission, traditionally attributed to *L. infantum* in the Old World and *L. chagasi* in the New World, belong to the same species, the reason why *L. chagasi* has been synonymized with *L. infantum* [3]–[5].

Studies concerning the analysis of genetic variability among *L. infantum* isolates from distinct geographic areas and reservoirs are of great clinical and epidemiological interest. Thus a high degree of intraspecific variability of *L. infantum* populations, with particular interest to Mediterranean strains, has been demonstrated according to biological [6], biochemical [7]–[9] and molecular [10], [11] parameters.

Several genomic and kinetoplast DNA (kDNA) targets have been employed to ascertain genetic variability of *L. infantum*, which include ribosomal transcribed-spacer and mini-exon regions [12], rRNA and antigen genes [13], [14], micro-satellites [5], [10], [15]–[18], in addition to alleatory genomic sites [10], [19]–[22].

Concerning the kDNA analysis, schizodeme has been commonly employed for genetic analysis of *L. infantum* parasites maintained in culture or laboratory animals [4], [20], [22]–[24] but few studies have performed a comparative analysis among *L. infantum* isolates originated from the Old as well as the New World [4], [5].

Low-stringency Single Specific Primer-PCR (LSSP-PCR) is a sensitive and reproducible technique for the identification of polymorphisms on DNA target fragments [25]. The production of kDNA signatures by LSSP-PCR is a two-step procedure in which the kDNA target is firstly amplified from the parasite minicircles network by using a conventional PCR protocol. In the second step the kDNA fragment previously obtained is submitted to amplification under low-stringency conditions with the employment of one of the specific primers so as to generate a profile composed by several bands which reflects the heterogeneity of the sequence under analysis [26]–[28]. The large number of SNPs (single nucleotide polymorphisms) present in the kDNA sequence, along with the variation observed in the number of minicircles classes in trypanosomatids, are responsible for generating, in each kDNA signature, DNA fragments of different sizes which can be considered as kDNA markers generated by LSSP-PCR [27], [28].

This technique has been efficiently employed to ascertain genetic variability in protozoa parasites, such as *Entamoeba histolytica* [24], *Trypanosoma cruz*i [27]–[33] and *Trypanosoma rangeli* [34]. However, despite of the effectiveness of LSSP-PCR to detect genetic variability in kinetoplastid parasites, only few studies have used LSSP-PCR for analyzing intraspecies polymorphisms in *Leishmania* [35]–[37].

This study reports the use of the LSSP-PCR technique for genetic characterization of *L. infantum* strains isolated from humans and dogs reservoirs from Portugal and Brazil. To our knowledge, this is the first study to employ LSSP-PCR for genetically comparing strains from both the Old and the New World. The intraspecific polymorphism of the parasite kDNA minicircle hypervariable region could be translated into specific and very informative kDNA signatures, which allowed the grouping of strains isolated from humans and dogs in specific clades. Thus, LSSP-PCR constitutes a simple and new molecular tool to perform genetic characterization of *L. infantum* strains isolated from distinct reservoirs and geographical regions.

## Methods

### Parasites

Forty *L. infantum* strains isolated from human patients with VL and canine reservoirs from Belo Horizonte, Minas Gerais state, Brazil and Lisbon, Portugal were used in this study (Table 1). Parasites were isolated from the bone marrow of patients as well as diseased dogs which lived in distinct neighborhoods of the urban areas of those cities, with no epidemiological links identifiable among the humans and dogs from which the strains were originated. Ten strains from each host and belonging to each geographical origin were evaluated. The brazilian strains were obtained from the *Leishmania* strains’ collection from the Universidade Federal de Minas Gerais and from the Centro de Pesquisas René Rachou – FIOCRUZ, Belo Horizonte, Minas Gerais, Brazil. Strains from Portugal were provided by the Unidade de Leishmanioses, Instituto de Higiene e Medicina Tropical, Universidade Nova de Lisboa, Lisbon, Portugal. All strains were grown at 25±1°C in α-MEM (Minimum Essential Medium; Gibco BRL-USA) medium supplemented with 10% fetal bovine serum, cryopreserved at the first passages after isolation, and towed exactly at the same time, before performing the simultaneous biochemical and molecular analysis. The strains were submitted to the zymodeme caracterization by analysis of ten methabolic isoenzymes. All parasites were characterized as *L. infantum* on the zymodeme MON-1 by using Multilocus Enzyme Electrophoresis (MLEE) analysis (data not shown).

**Table 1:** *Leishmania infantum* strains used in this study.

*Leishmania infantum* strains isolated from Brazil#			
Strain	International code	Origin reservoirs - Code	kDNA genotype§
BH 46	MHOM/BR/1970/BH 46	Human – HB1	I
RR072	MHOM/BR/2001/RR072	Human – HB2	I
RR055	MHOM/BR/1998/RR055	Human – HB3	III
RR059	MHOM/BR/1996/RR059	Human – HB4	IV
RR011	MHOM/BR/2001/RR011	Human – HB5	I
RR050	MHOM/BR/1996/RR050	Human – HB6	III
RR053	MHOM/BR/1998/RR053	Human – HB7	II
RR058	MHOM/BR/1996/RR058	Human – HB8	VII
RR056	MHOM/BR/1999/RR056	Human – HB9	I
RR054	MHOM/BR/1998/RR054	Human – HB10	III
RR087	MCAN/BR/2000/RR087	Canine – CB1	I
RR065	MCAN/BR/1996/RR065	Canine – CB2	I
BH401	MCAN/BR/2002/BH401	Canine – CB3	V
RR061	MCAN/BR/1996/RR061	Canine – CB4	VII
RR069	MCAN/BR/1996/RR069	Canine – CB5	IV
BH402	MCAN/BR/2002/BH402	Canine – CB6	IV
BH403	MCAN/BR/2002/BH403	Canine – CB7	II
BH400	MCAN/BR/2002/BH400	Canine – CB8	I
BH406	MCAN/BR/2002/BH406	Canine – CB9	I
RR066	MCAN/BR/1996/RR066	Canine – CB10	VI
***Leishmania infantum*** ** strains isolated from Portugal** *			
**Strain**	**International code**	**Origin reservoirs - Code**	**kDNA genotype**
IMT 245	MHOM/PT/1998/IMT245	Human – HP1	II
IMT 144	MHOM/PT/1998/IMT144	Human – HP2	I
IMT 242	MHOM/PT/1998/IMT242	Human – HP3	VIII
IMT 248	MHOM/PT/1999/IMT248	Human – HP4	V
IMT 249	MHOM/PT/1999/IMT249	Human – HP5	VI
IMT 224	MHOM/PT/1996/IMT224	Human – HP6	III
IMT 233	MHOM/PT/1997/IMT233	Human – HP7	I
IMT 225	MHOM/PT/1997/IMT225	Human – HP8	IX
IMT 226	MHOM/PT/1997/IMT226	Human – HP9	IV
IMT 234	MHOM/PT/1997/IMT234	Human – HP10	II
IMT 254	MCAN/PT/1998/IMT242	Canine – CP1	I
IMT 229	MCAN/PT/1997/IMT229	Canine – CP2	III
IMT 256	MCAN/PT/1999/IMT256	Canine – CP3	I
IMT 262	MCAN/PT/2000/IMT 262	Canine – CP4	III
IMT 339	MCAN/PT/2003/IMT339	Canine – CP5	II
IMT 321	MCAN/PT/2003/IMT321	Canine – CP6	IV
IMT 322	MCAN/PT/2003/IMT322	Canine – CP7	II
IMT 326	MCAN/PT/2003/IMT326	Canine – CP8	III
IMT 319	MCAN/PT/2003/IMT319	Canine – CP9	V
IMT 320	MCAN/PT/2003/IMT320	Canine – CP10	I

WHO Reference strains			
Strain	International code	Species	kDNA genotype
PPT5	MHOM/BR/1974/PP75	*Leishmania* (*Leishmania*) *infantum*	V
IPT1	MHOM/TN/1980/IPT1	*Leishmania* (*Leishmania*) *infantum*	VIII

# Parasite strains isolated from Belo Horizonte, Minas Gerais State, Brazil;

* Strains isolated from the Lisbon Metropolitan Region (LMR), Portugal;

§ KDNA genotypes were determined by LSSP – PCR technique by using the MC1 primer as the driver.

### DNA Extraction and PCR Amplification

Total DNA was isolated from 10^9^ promastigotes cultures by using a conventional protocol based on the Proteinase K digestion, phenol/chloroform/isoamyl alcohol extraction and NaOAc/ethanol precipitation steps. Amplification of a 447 bp kDNA minicircle fragment was carried out with MC1 (5′-GTTAGCCGATGGTGGTCTTG-3′) and MC2 (5′-CACCCATTTTTCCGATTTTG-3′) primers, which amplify the hypervariable region of *L. infantum* kDNA minicircle. PCR was performed as previously described, by using 10 ng of parasite DNA and the kit *PureTaq Ready to go PCR Beads* (Amersham Biosciences) [38]. Five microliters of PCR products were analyzed on silver-stained 6% polyacrylamide gels.

### LSSP-PCR Analysis

For the production of kDNA signatures from the 447 bp *L. infantum* kDNA fragments, PCR products were purified by using the kit Wizard® PCR Preps – DNA purification System (Promega). After 10 times dilution in deionized water, 2 µL of PCR-purified products were used as template in a second amplification step under low stringency conditions by using the MC1 primer as driver. LSSP-PCR profiles were visualized on silver-stained 6% polyacrylamide gel as previously described [25], [27]. To assure reproducible results, all reactions were performed at least in two independent experiments. In order to evaluate the banding pattern obtained to each strain and to define each genotype, the gels were carefully analyzed to determine the size of the main fragments of LSSP-PCR profiles. To perform this analysis the LabImage-1D Gel Analysis software, Version 2.7.2 (Copyright 1999–2004, Kapelan GmbH, Halle, Saale, Germany), available at the website www.labimage.net, was employed. This software is able to determine the size of each DNA fragment (band) selected in the gel constitutive of each kDNA signature in comparison with the fragments of the 1 Kb DNA Ladder (Invitrogen-BRL).

### Phenetic Analysis

Strong bands ranging in size from 93 to 1619 bp and more clearly visible were selected for phenetic analysis. LSSP-PCR bands were compared using the simple matching coefficient of similarity to determine the proportion of mismatched bands between pairs of isolates. The similarity matrix was transformed into a dendrogram using the UPGMA algorithm [39]. Phenetic analysis was performed with the NTSYS-pc program (version 2.02, Exeter Software, Setauket, NY, USA).

### DNA Sequencing

To determine the nucleotide sequence of the 447 bp *L. infantum* kDNA-PCR products, eight DNA samples obtained from Brazilian strains and eight DNA samples isolated from Portugal strains were randomly selected and the kDNA-PCR products were submitted to DNA sequencing, in addition to the PCR products amplified from the two WHO reference strains (PP75 and IPT1). PCR products were purified with the kit Wizard® PCR Preps – DNA purification System (Promega) and sequenced by using the Big Dye (Cycle Sequencing) Terminator Kit. Nucleotide sequences were obtained by the Automated Sequencing System ABI 3130 and edited with the Sequence Scanner software V1.0 (Applied Biosystems). The alignment of kDNA sequences from the isolates with those obtained from the reference strains was performed with the CLUSTALW software package (EMBL-EBI) (http://www.ebi.ac.uk/clustalw/). The sequences were analyzed for homology search by using the BLAST-N (Basic Alignment Search Tool) avaiable at the http://www.ncbi.nlm.nih.gov/BLAST 3.2.1. All new data have been deposited in Gene Bank (Accession numbers GenBank JQ609524-JQ609541).

### Ethic Statments

Research in this study was subjected to ethical review by the European Commission and approved as part of the contract negotiation for the Project LeishEpinetSA (contract 01547); this work was carried under all the relevant European regulations. Research was also approved by the ethics committees of the Universidade Federal de Minas Gerais, Belo Horizonte, Minas Gerais and of the FIOCRUZ, Rio de Janeiro, Brazil. In all cases the *Leishmania* parasites were isolated from human patients as part of the normal diagnosis and treatment, and from dogs as part of the disease control measurements, under the written consent recorded at the time of clinical examination. Data on isolates were coded and anonymized.

## Results

In the present work, the genetic characterization of forty *L. infantum* strains, ten isolated from each distinct reservoir accounting for 20 strains from each geographical region, was performed. Data from the partial (410 bp) DNA sequencing of the 447 bp *Leishmania* kDNA region analyzed in this study, which had previously been amplified from 16 *L. infantum* strains (8 from humans and 8 from dogs), revealed a certain level of genetic polymorphism (Figure S1). Eight single nucleotide changes were identified, with two of these point mutations (at the positions 85 and 94) present only in the reference strains but absent in *L. infantum* isolates originated from both human and canine reservoirs. Interestingly, some polymorphic sites were related to the geographical origin of the strains. For example, an A to G transition (at the position 91) was verified in the majority of *L. infantum* samples from Portugal. This polymorphism was also observed in the totality of *L. infantum* strains (at the positions 149, 262, 297), isolated either from human or canine reservoirs. Regarding the intra-specific polymorphisms related to the reservoir origin from the strains, one polymorphism present only in the kDNA sequences from strains isolated from human VL cases from Portugal was identified (a T to G transversion, at the position 220). In addition to this host origin site, this point mutation was also present in the minicircle sequence from strains isolated from canine reservoirs isolated from Brazil.

**Figure 1:**
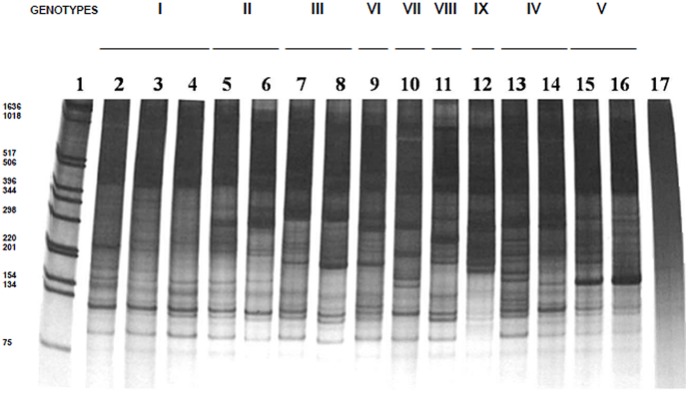
KDNA signatures of the 447-bp minicircle fragment of *L. infantum* (* = L. chagasi*) strains isolated from human patients. Five µ l of the LSSP-PCR reaction products, were loaded in each lane of a 6% polyacrylamide gel and silver stained. Lanes 2 to 17: genotype I (lanes 2–4: HP7, HB5 and HB9); genotype II (lanes 5–6: HP10 and HP1); genotype III (lanes 7–8: HP6 and HB6); genotype VI (lane 9: HP5); genotype VII (lane 10: HB8); genotype VIII (lane 11: HP3); genotype IX (lane 12: HP8); genotype IV (lanes 13–14: HB4 and HB9); genotype V (lanes 15–16: HP4 and PP75); lane 17 the negative control of the LSSP-PCR reaction. Migration of the markers of the 1 Kb DNA ladder (Life Technologies, Inc., Gaithersburg, MD) is shown in lane 1, with the following molecular sizes (from the bottom up): 75, 134, 154, 201, 220, 298, 344, 396, 506, 517, 1018 and 1636 bp. Genotypes are indicated by roman numbers.

**Figure 2:**
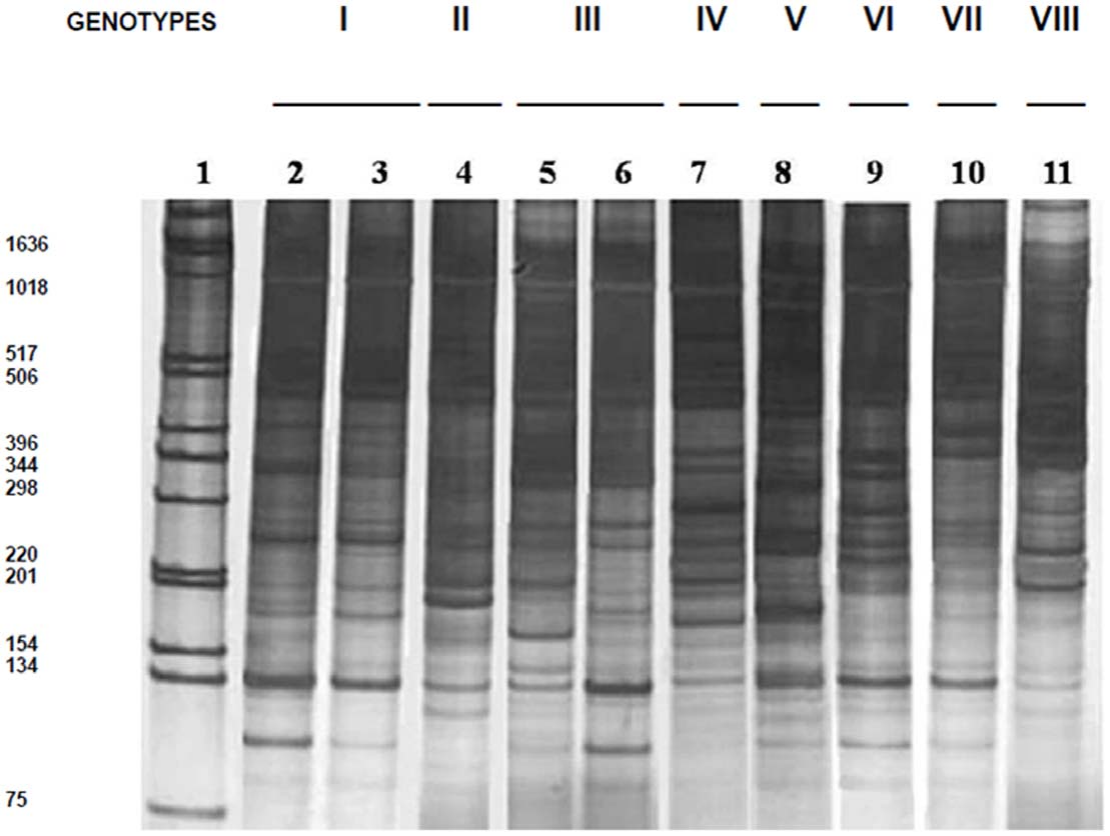
KDNA signatures of the 447-bp minicircle fragment of *L. infantum* (* = L. chagasi*) strains isolated from the canine reservoir. Five µ l of the LSSP-PCR reaction products, were loaded in each lane of a 6% polyacrylamide gel and silver stained. Lanes 2 to 11: genotype I (lanes 2–3: CB2 and CP3); genotype II (lane 4: CP5); genotype III (lanes 5–6: CB7 and CP2); genotype IV (lane 7: CP6); genotype V (lane 8: CB3); genotype VI (lane 9: IPT1); genotype VII (lane 10: CB10); genotype VIII (lane 11: CB4). Migration of the markers of the 1 Kb DNA ladder (Life Technologies, Inc., Gaithersburg, MD) is shown in lane 1, with the following molecular sizes (from the bottom up): 75, 134, 154, 201, 220, 298, 344, 396, 506, 517, 1018 and 1636 bp. Genotypes are indicated by roman numbers.

Electrophoretic multiple-banded profiles resulting from the 447 bp kDNA LSSP-PCR analysis of 40 strains isolated from Brazil and Portugal and two reference strains were generated with the MC1 primer (Figures 1 and 2). In order to investigate one possible existing association among the kDNA genetic profiles and the strains’ reservoir type or geographic origin, a comparative analysis among kDNA signatures obtained from *L. infantum* isolates originated from the same host origin (human or canine), but from distinct geographic areas, was performed.

Very informative profiles were obtained by LSSP-PCR analysis of kDNA PCR products spaning the hypervariable region previously amplified from isolates originated from human VL cases (Figure 1). Genetic signatures were composed by DNA fragments varying from 93 to 1619 bp and exhibited a high intraspecific variability, even though LSSP-PCR profiles obtained from distinct strains were able to share several DNA fragments (Table S1). For example, most LSSP-PCR patterns shared, at least partially, several groups of bands, such as the triplets (1619, 564 and 510 bp), (264, 242 and 226 bp) and (154, 141 and 132 bp) - (Table S1). By comparing the size (in base pairs) and the number of the main DNA fragments of each kDNA signature, it was possible to determine the similarity existing among LSSP-PCR patterns obtained from distinct human strains, which were then grouped in nine genotypes (I to IX; Figure S1 and Table S1). Among the LSSP-PCR patterns obtained from human isolates, the I, II and III genotypes were able to group a higher number of strains (6, 3, and 4, respectively), followed by the genotypes IV and V (Table S1).

The LSSP-PCR profiles obtained by analysis of *L. infantum* kDNA hypervariable region of parasites isolated from canine reservoirs also exhibited a high level of DNA polymorphisms (Figure 2). The similarity observed among kDNA signatures obtained from distinct canine strains allowed to group the isolates in eight genotypes (I to VIII, Figure 2), which were mainly composed by DNA fragments ranging from 108 to 1435 bp (Table S1). Genotype I was able to group the highest number of canine strains (6), followed by genotypes III and IV which grouped 4 isolates each (Table S1). Furthermore, a partial or complete band sharing among distinct kDNA patterns obtained from canine isolates was performed.

**Figure 3:**
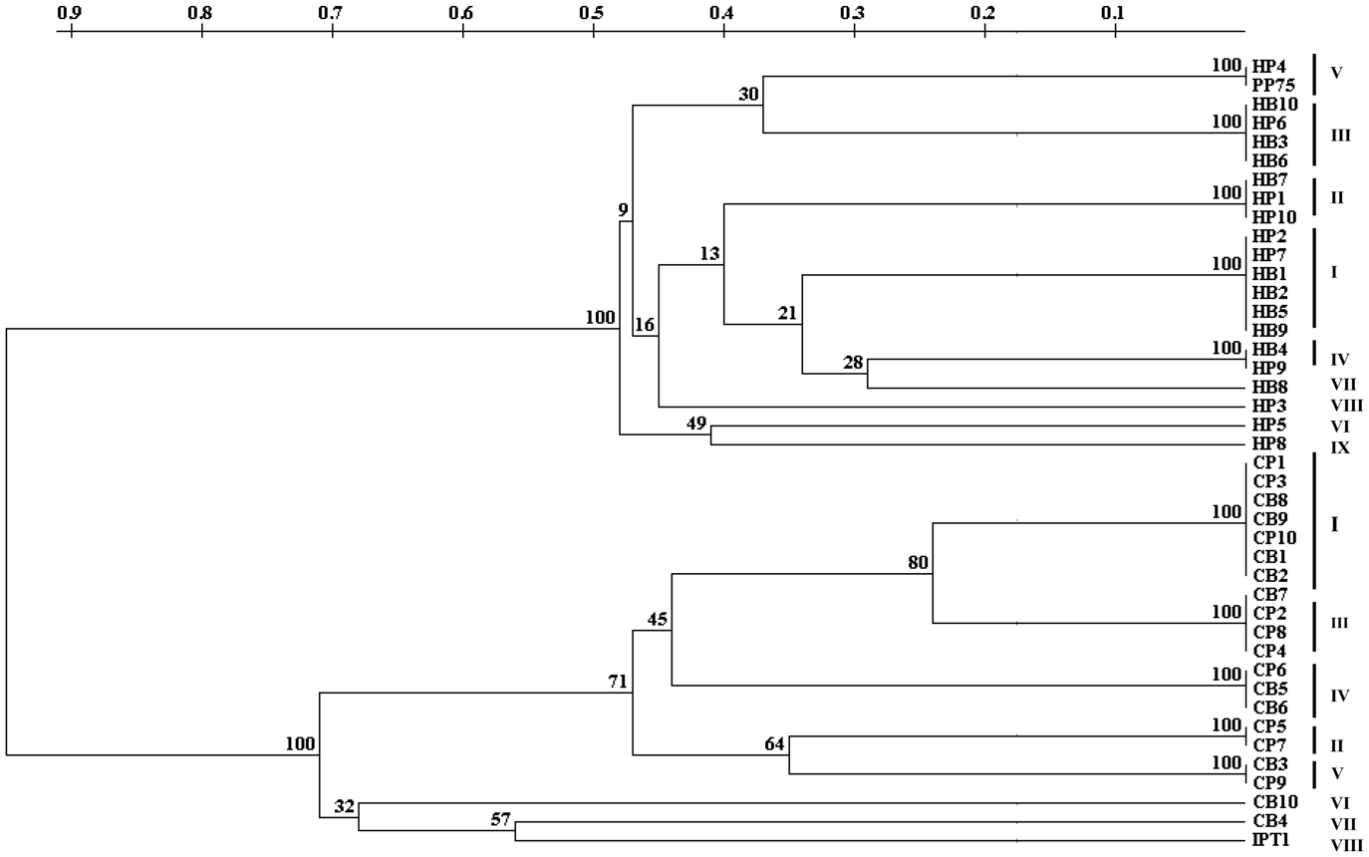
Dendogram obtained by analysis of LSSP-PCR profiles of 40 *Leishmania infantum* (* = L. chagasi*) strains isolated from VL human cases or canine reservoirs. Genetic distances obtained by LSSP-PCR analysis of the 447 Pbp kDNA minicircle. The main constitutive bands of the LSSP-PCR profiles were used to build the phenetic tree by using the UPGMA method. Strains belonged to the same kDNA genotype are indicated on the right.

The main constitutive bands of *L. infantum* kDNA signatures obtained from human cases and canine reservoirs were analyzed in conjunction and employed to build a phylogenetic tree (Figure 3), where strains isolated from distinct hosts were grouped in two main branches, A and B. The nine genotypes obtained from human isolates (branch A) were grouped in two subgroups with the majority of kDNA patterns (genotypes I, II, III, IV, V, VII and VIII) grouped in the upper subgroup. The eight kDNA signatures obtained from canine isolates (branch B) were also assembled in two clusters, with the highest number of profiles (genotypes I, II, III, IV and V) and isolates (17) grouped within the lower cluster. The dendogram basal nodes which distinguished genotypes obtained from human and canine strains were statistically significant (bootstrap values of 99 and 100%).

## Discussion

Despite the existence of a number of previously developed studies concerning the *L. donovani* complex the epidemiology of VL is still poorly known, specially when its medical importance and impact are considered.

Several methods have been employed to examine genetic polymorphisms within the *L. donovani* complex and into each species. Multilocus Enzyme Electrophoresis (MLEE) analysis of *L. infantum* ( = *L. chagasi*) strains demonstrated a wide enzymatic polymorphism within this species, even though the zymodeme MON-1 has been predominantly found in all the endemic areas and in both human and canine reservoirs [5], [8], [9], [15], [40], [41].

However, due to their sensitivity, stability, reproducibility and genetic resolution power, molecular techniques have preferentially been used to ascertain genetic variability in *L*. *infantum* (reviewed in references [42]–[44]). Concerning the studies focused on the kDNA target, the RFLP’s analysis of *L. infantum* strains isolated from dogs, humans or sand flies and originated from Portugal [24], Spain [10], Italy [45], Israel [46] and Brazil [47], a high level of genetic heterogeneity has been demonstrated among the isolates and a weak correlation between the RFLP’s profiles and the strains’ geographical or reservoir origin was observed [10], [24], [46].

Molecular methodologies based on the analysis of *Leishmania sp.* kDNA minicircles, such as RFLP’s and LSSP-PCR, exhibit several advantages when compared with techniques focused on the investigation of nuclear targets as a higher sensitivity and polymorphism content [48]–[51]. However, the number of DNA fragments and the informativeness of the kDNA-RFLP’s profiles are dependent on the number of the restriction enzyme employed [24]. In this sense, LSSP-PCR can be comparatively considered a simpler and more sensitive molecular tool for ascertaining genetic variability in *Leishmania sp.* since the kDNA signatures obtained in this and in previous studies, from analysis of *L. infantum* and *L. braziliensis* [35], [37] strains, respectively, were very informative and composed of multiple fragments obtained in a single LSSP-PCR step, without the employment of DNA probes or restriction enzymes [10], [24], [45]–[46].

Few studies have performed a comparative analysis among *L. infantum* strains isolated from both the Old and the New World, and the present study is the first to employ LSSP-PCR for this purpose. The kDNA signatures exhibited a significant level of association with the host type (human and canine), but no correlation between the genotypes and the isolates’ geographical origin could be observed. Similar results were previously reported by using kDNA – RFLP’s technique [10], [24], [46].

It can be presumed that the heterogeneity of gene signatures obtained by LSSP-PCR analysis of *L. infantum* kDNA minicircles could reflect not only the large genetic diversity frequently observed in strains from the *Leishmania sp.* genus, but also the high intraspecific variability existing among kDNA molecules from the trypanosomatids *Leishmania* (reviewed in [48], [50]) and *Trypanosoma cruzi* [51], [52]. LSSP-PCR largely depends on the presence of SNPs (single nucleotide polymorphisms) in the target DNA fragment in addition to the sequence number under analysis. Concerning the kDNA analysis, a high mutation rate is frequently observed in the trypanosomatids kDNA hypervariable region [52]. Additionally, kDNA minicircles from *Leishmania* species, which are present at thousands of copies per cell, are about 750 bp to 1 Kb in size, contain one 250 bp-conserved region and one 500–760 bp variable region which codifies one single 70 bp-guide RNA (gRNA) gene [50], [53]. Minicircles form a heterogeneous group of DNA sequences depending on the species; large number of said minicircles has been explained by those different minicircle-encoded gRNA required by the RNA editing [50], [51], [53]. It is assumed that LSSP-PCR targeting kDNA minicircles produce profiles that reflect polymorphisms of the predominant classes of minicircles in each *Leishmania* parasite, thus producing specific and distinct LSSP-PCR multi-banded patterns, as prevously suggested for the kDNA genetic signatures obtained for the species *L.* *braziliensis* [35]–[37]. RFLP’s analysis of the *Leishmania sp.* kDNA hypervariable region also demonstrated a considerable level of genetic variability, indicating that even closely related *Leishmania* strains could exhibit several types of minicircles molecules [49], [50], [53].

The sequence analysis of the 447 bp kDNA hipervariable region from the *L. infantum* isolates revealed the presence of some nucleotide substitutions, which were shared among strains isolated from the same geographical origin or host type.It can therefore be expected that distinct *L. infantum* kDNA gene signatures could have been generated by the presence of these polymorphisms in the kDNA target fragments, as it has previously been demonstrated by the LSSP-PCR analysis of similar hypervariable human mitochondrial [54] or parasite kinetoplast [27], [28], [35]–[37] DNA targets. However, since the LSSP-PCR technique is potentially able to target the main part of kDNA fragments composing the 447 bp product used as template for LSSP-PCR reaction, it is likely that the majority of kDNA sequences possibly probed by LSSP-PCR, and which contributed to the formation of *L. infantum* kDNA signatures, were not detected by the sequencing approach employed in the present study.

The genetic diversity of *L. donovani* complex populations was extensively investigated by means of DNA sequencing analysis of 10 enzyme-coding genes, which was able to generate aproximately 12,000 genomic characteres (or polymorphic sites) [40]. While the highest haplotype diversity was observed among african *L. donovani* strains, the nucleotide and haplotype variety was lower in *L. infantum* populations isolated from Europe, where six out of 14 strains shared the same MLEE type (MON-1) and the same 10 enzyme genes-haplotype [40]. The identification of molecular markers capable of discriminating among distinct MON-1 strains is crucial for a better understanding of the population structure and potential spreading of *L. infantum* virulent VL strains within Europe and South America [10], [19], [22]. In this aspect LSSP-PCR can be considered an useful molecular tool to identify genetic variability into MON-1 zymodeme strains, since the analysis of 40 strains isolated from human and canine VL cases has allowed to obtain 9 and 8 distinct *L. infantum* genotypes, respectively.

The evolutionary history of the *L. donovani* complex in the New World is still under debate. The hypothesis considered as the most plausible suggests that *L. infantum* parasites could have been brought from Europe to America during historical times [55] by humans and specific reservoirs as dogs, originating the *L. infantum* strains circulating at present days [40], [55]. This view has received support from important studies performed either in the last two decades [4], [7], [56]–[58] or recently [5], [40]. A microsatellite-comparative analysis of *L. infantum* strains isolated from different foci from both the Old and the New World identified two distinct sub–populations (1 and 2), with the majority of the New World strains (including the Brazilian ones) belonging to the Old World MON-1 or population 1-type. Therefore, the last study revealed the recent Old World-origin of the New World *L. infantum* populations, but no correlation among the *L. infantum* genotypes and the clinical picture or host background was found [5].

No association among the kDNA signatures and the RFLP’s profiles with the geographical origin of *L. infantum* strains was observed in this as well as in previous studies [10], [24], [46]. A new taxonomy for the *L. donovani* complex was recently proposed in a study based on a multi-factorial genetic analysis of 25 geographically representative strains (96% isolated from human cases): the *L. donovani* and *L. infantum* would be the only recognized species within the *L. donovani* complex [40]. The isolates were phylogenetically grouped in two main clusters (*L. donovani* – strains from East Africa, India and Middle East; *L. infantum* – parasites from Europe, North Africa and Central America) strongly correlated with geographical (continental) origin, but not with clinical outcome or current taxonomy [40]. The taxonomy and phylogeny proposed were consistent with geographical distribution of *L. donovani* and *L. infantum* [40]. However, it is important to mention that our analysis was restricted to strains from Portugal (Europe) and Brazil (South America), which are phylogenetically related to the same cluster of *L. infantum* species [40].

Furthermore, the relative lack of association observed in our study among the kDNA genotypes and the geographical origin of *L. infantum* isolates could be due to the epidemiological differences verified between the visceral leishmaniasis in Brazil and Portugal. Concerning the vectors, two distinct genera are involved in the VL transmission in these countries-*Phlebotomus perniciosus* and *P. ariasi* (Subgenus *Larroussius*) in Portugal [59] and *Lutzomyia longipalpis* and *L. cruzi* (Subgenus *Lutzomyia*) in Brazil [60]. Other relevant epidemiological contrasts include the environment, the climate and the occurrence of the risk factors associated with this disease.

Both Belo Horizonte and Lisbon are scenario for urban VL transmission with the disease in rapid urbanization due to the increasing incidence of the domestic reservoirs-infected dogs [61]. Currently, approximatelly 10% of all dogs exhibit positive test for canine VL and this high observed seroprevalence in animals from Belo Horizonte represents a leading risk factor for introducing the disease to the human population [62]. Additionally, the global climate changes, associated with a higher density and activity of sand flies during a larger period, might enhance the number of days favorable for transmission of parasites to humans and animals with a concomitant increase of incidence [61]. Therefore the certain level of association among the kDNA genotypes and the host type origin observed in this study was probably related with the fact that all the *L. infantum* populations analyzed were isolated from urban areas of big cities such as Belo Horizonte and Lisbon, where the main aspects of VL transmission are very similar, and the canine reservoir plays an important role in the disease dissemination.

It is important to emphasize that a relatively small number of strains originated from the two distinct geographical regions, Brazil and Portugal, were analyzed in the present study. Taking the consideration the extensive area comprising the Brazilian territory, it is reasonable to suppose that the isolates here analyzed could not be representative of the whole country. However, as previously mentioned, the strains analyzed in this work were isolated from urban centers of big cities which share similar epidemiology characteristics for the endemic transmission of the visceral leishmaniasis [59]–[62], regardless of the distinct geographical localization of these endemic sites in said countries. For this reason the results obtained in this study are still relevant since strains isolated from both the Old and the New World were genetically analyzed by LSSP-PCR in the context of the reservoir origin, even though the limitations concerning the small sample size analyzed can not be discarded.

It should also be noted that the *Leishmania* strains analyzed in this study were collected over a long period of time, specially the Brazilian isolates ones. However in the last 30 years our laboratory has achieved much experience in parasite isolation and handling procedures in order to rigorously maintain the biological and biochemical characteristics of each strain under all experimental procedures. These precautions have allowed us to reach reproducible and confident results in previous studies developed on the genetic variability of *Leishmania sp*. by using DNA fingerprinting and RAPD approaches [63], [64], [65].

In addition to the present work, three other studies have used LSSP-PCR for the analysis of kDNA minicircles from parasites from the *Leishmania* genus, but they were focused on the evaluation of *L. braziliensis* parasites maintained in culture [36] or present in biological samples, such as active lesions or scars [35], [37]. The LSSP-PCR profiles obtained in those studies seemed to be less informative than the kDNA gene signatures obtained in this work, but in all three studies the LSSP-PCR technique was able to reveal intraspecific variation of *L. braziliensis* isolates [35]–[37]. Moreover, an association between the kDNA genotypes and the clinical course [36] or the presence of specific subpopulations [35] could be satisfactorily demonstrated for the tegumentary leishmaniasis.

This work describes the usefulness of LSSP-PCR technique when performing genetic typing of *L. infantum* strains originated from the Old as well as the New World and from human and canine hosts affected by visceral leishmaniasis. Very informative and multi-banded kDNA signatures were obtained, which were associated with the polymorphisms present at the kDNA hypervariable sequence with some *L. infantum* kDNA genotypes exhibiting a correlation with the origin reservoir. In addition, a higher level of variability was observed in the *L. infantum* kDNA signatures of strains isolated from human patients, either from Brazil or Portugal, in comparison to those profiles obtained from canine isolates, whose strains seemed to exhibit a lesser level of intraspecific kDNA polymorphism. In summary, we consider that the LSSP-PCR technique employed in the present work represents a simple and new molecular tool to perform genetic characterization of *L. infantum* isolates. Its employment can effectively contribute for a better epidemiology and clinical understanding of the visceral leishmaniasis and also provide opportunities to advise health authorities about the most effective measures for prevention and control of this parasitic disease.

## Supporting Information

Figure S1 **Alignment of the 447 bp** ***L. infantum*** **(** ***= L. chagasi*** **) kDNA minicircle sequences obtained for two reference strains, PPT5 and IPT1, and ten** ***L. infantum*** **strains, isolated from human and canine hosts from Brazil and Portugal.** Bases labeled in gray indicate polymorphisms found only in the sequence of Reference strains. Bases marked in yellow indicate polymorphisms related with the strains’ geographic origin, while bases marked in red indicate polymorphic sites related to both, the geographic and reservoir origin of the analyzed strains. The strains codes are indicated on the left.
(DOC)

Table S1 **Relative size in base pairs of main DNA fragments of the 447 bp kDNA minicircle fragment LSSP-PCR genotypes from** ***Leishmania infantum*** **strains using MC1 primer.** The bands shared among the kDNA signatures are marked in gray.
(DOC)
