# Supplementary material for: KDNA Genetic Signatures Obtained by LSSP-PCR Analysis of Leishmania (Leishmania) infantum Isolated from the New and the Old World
Source: PLoS One. 2012 Aug 17;7(8):e43363. doi: 10.1371/journal.pone.0043363 (PMC3422226; doi:10.1371/journal.pone.0043363)
Supplement: Figure S1 — Alignment of the 447 bp L. infantum ( = L. chagasi ) kDNA minicircle sequences obtained for two reference strains, PPT5 and IPT1, and ten L. infantum strains, isolated from human and canine hosts from Brazil and Portugal. Bases labeled in gray indicate polymorphisms found only in the sequence of Reference strains. Bases marked in yellow indicate polymorphisms related with the strains’ geographic origin, while bases marked in red indicate polymorphic sites related to both, the geographic and reservoir origin of the analyzed strains. The strains codes are indicated on the left. (DOC) [file pone.0043363.s001.doc]

| | **Code** | IPT1 | 10 20 30 40 50 60 70 80 90  TTCTCTTTAT ATTGATATTA AAGGTAAGTG CTTGGACATA TGATATAGCC ATAGCGCTTT AGAATAGTTC GACTCCGAAG ACCCGGTCTG | | --- | --- | --- | |  | PP75 | .......... .......... .......... .......... .......... .......... .......... .......... ....A..... | | CP1 | IMT254 | .......... .......... .......... .......... .......... .......... .......... .......... ....A..... | | CP4 | IMT262 | .......... .......... .......... .......... .......... .......... .......... .......... ....A..... | | CP5 | IMT339 | .......... .......... .......... .......... .......... .......... .......... .......... ....A..... | | CP7 | IMT322 | .......... .......... .......... .......... .......... .......... .......... .......... ....A..... | | HP2 | IMT144 | .......... .......... .......... .......... .......... .......... .......... .......... ....A..... | | HP6 | IMT224 | .......... .......... .......... .......... .......... .......... .......... .......... ....A..... | | HP8 | IMT225 | .......... .......... .......... .......... .......... .......... .......... .......... ....A..... | | HP9 | IMT226 | .......... .......... .......... .......... .......... .......... .......... .......... ....A..... | | CB1 | RR066 | .......... .......... .......... .......... .......... .......... .......... .......... ....A..... | | CB2 | RR065 | .......... .......... .......... .......... .......... .......... .......... .......... ....A..... | | CB4 | RR061 | .......... .......... .......... .......... .......... .......... .......... .......... ....A..... | | CB6 | BH402 | .......... .......... .......... .......... .......... .......... .......... .......... ....A..... | | HB2 | RR072 | .......... .......... .......... .......... .......... .......... .......... .......... ....A..... | | HB3 | RR055 | .......... .......... .......... .......... .......... .......... .......... .......... ....A..... | | HB4 | RR059 | .......... .......... .......... .......... .......... .......... .......... .......... ....A..... | | HB6 | RR050 | .......... .......... .......... .......... .......... .......... .......... .......... ....A..... | | **Code** | IPT1 | 100 110 120 130 140 150 160 170 180  AGGCAGTGTT AAGTATACAT TAATCTAGTA TATAATTTAG CATATAATAA CTGACATTAC TCGTACACTA TAAGTATTAT GTTTAATATA | |  | PP75 | A..C...... .......... .......... .......... .......... ...A....A. .......... .......... .......... | | CP1 | IMT254 | A..T...... .......... .......... .......... .......... ...A....A. .......... .......... .......... | | CP4 | IMT262 | G..T...... .......... .......... .......... .......... ...A....A. .......... .......... .......... | | CP5 | IMT339 | A..T...... .......... .......... .......... .......... ...A....A. .......... .......... .......... | | CP7 | IMT322 | G..T...... .......... .......... .......... .......... ...A....A. .......... .......... .......... | | HP2 | IMT144 | G..T...... .......... .......... .......... .......... ...A....A. .......... .......... .......... | | HP6 | IMT224 | G..T...... .......... .......... .......... .......... ...A....A. .......... .......... .......... | | HP8 | IMT225 | G..T...... .......... .......... .......... .......... ...A....A. .......... .......... .......... | | HP9 | IMT226 | G..T...... .......... .......... .......... .......... ...A....A. .......... .......... .......... | | CB1 | RR066 | A..T...... .......... .......... .......... .......... ...A....G. .......... .......... .......... | | CB2 | RR065 | A..T...... .......... .......... .......... .......... ...A....G. .......... .......... .......... | | CB4 | RR061 | A..T...... .......... .......... .......... .......... ...A....G. .......... .......... .......... | | CB6 | BH402 | A..T...... .......... .......... .......... .......... ...A....G. .......... .......... .......... | | HB2 | RR072 | A..T...... .......... .......... .......... .......... ...A....G. .......... .......... .......... | | HB3 | RR055 | A..T...... .......... .......... .......... .......... ...G....G. .......... .......... .......... | | HB4 | RR059 | A..T...... .......... .......... .......... .......... ...G....G. .......... .......... .......... | | HB6 | RR050 | A..T...... .......... .......... .......... .......... ...A....G. .......... .......... .......... | | **Code** | IPT1 | 190 200 210 220 230 240 250 260 270  TTGCTGTAGT ATGTATTTGT GTGCTGTGTG TAGTAGTAAT ATCTATACCG ATATATTTAT AGGTTGGCGC ATACTGCAGT GAATTGAAAA | |  | PP75 | .......... .......... .......... .........T .......... .......... .......... .......... .A........ | | CP1 | IMT254 | .......... .......... .......... .........T .....TACCG .......... .......... .......... .A........ | | CP4 | IMT262 | .......... .......... .......... .........T .......... .......... .......... .......... .A........ | | CP5 | IMT339 | .......... .......... .......... .........T .......... .......... .......... .......... .A........ | | CP7 | IMT322 | .......... .......... .......... .........T .......... .......... .......... .......... .A........ | | HP2 | IMT144 | .......... .......... .......... .........G .......... .......... .......... .......... .A........ | | HP6 | IMT224 | .......... .......... .......... .........G .......... .......... .......... .......... .A........ | | HP8 | IMT225 | .......... .......... .......... .........G .......... .......... .......... .......... .A........ | | HP9 | IMT226 | .......... .......... .......... .........G .......... .......... .......... .......... .A........ | | CB1 | RR066 | .......... .......... .......... .........T .......... .......... .......... .......... .G........ | | CB2 | RR065 | .......... .......... .......... .........G .......... .......... .......... .......... .G........ | | CB4 | RR061 | .......... .......... .......... .........G .......... .......... .......... .......... .G........ | | CB6 | BH402 | .......... .......... .......... .........G .......... .......... .......... .......... .G........ | | HB2 | RR072 | .......... .......... .......... .........T .......... .......... .......... .......... .G........ | | HB3 | RR055 | .......... .......... .......... .........T .......... .......... .......... .......... .A........ | | HB4 | RR059 | .......... .......... .......... .........T .......... .......... .......... .......... .A........ | | HB6 | RR050 | .......... .......... .......... .........T .......... .......... .......... .......... .G........ | | **Code** | IPT1 | 280 290 300 310 320 330 340 350 360  TTAATGAATT GGGGTCGGGC TGTGGGAAGG TGTCGTAAAT TCTGGAAAAT GATGGAAAAT GGCCAAAAAT GGGGGGAAAT TCCAAACTTT | |  | PP75 | .......... .......... ......A... .......... .......... .......... .......... .......... .......... | | CP1 | IMT254 | .......... .......... ......A... .......... .......... .......... .......... .......... .......... | | CP4 | IMT262 | .......... .......... ......A... .......... .......... .......... .......... .......... .......... | | CP5 | IMT339 | .......... .......... ......A... .......... .......... .......... .......... .......... .......... | | CP7 | IMT322 | .......... .......... ......A... .......... .......... .......... .......... .......... .......... | | HP2 | IMT144 | .......... .......... ......A... .......... .......... .......... .......... .......... .......... | | HP6 | IMT224 | .......... .......... ......A... .......... .......... .......... .......... .......... .......... | | HP8 | IMT225 | .......... .......... ......A... .......... .......... .......... .......... .......... .......... | | HP9 | IMT226 | .......... .......... ......A... .......... .......... .......... .......... .......... .......... | | CB1 | RR066 | .......... .......... ......G... .......... .......... .......... .......... .......... .......... | | CB2 | RR065 | .......... .......... ......G... .......... .......... .......... .......... .......... .......... | | CB4 | RR061 | .......... .......... ......G... .......... .......... .......... .......... .......... .......... | | CB6 | BH402 | .......... .......... ......G... .......... .......... .......... .......... .......... .......... | | HB2 | RR072 | .......... .......... ......G... .......... .......... .......... .......... .......... .......... | | HB3 | RR055 | .......... .......... ......G... .......... .......... .......... .......... .......... .......... | | HB4 | RR059 | .......... .......... ......G... .......... .......... .......... .......... .......... .......... | | HB6 | RR050 | .......... .......... ......G... .......... .......... .......... .......... .......... .......... | | **Code** | IPT1 | 370 380 390 400 410  TCTGGTCCTC CGGGTAGGGG CGTTCTGCAA AATCGGAAAA ATGGGTGA | |  | PP75 | .......... .......... .......... .......... ........ | | CP1 | IMT254 | .......... .......... .......... .......... ........ | | CP4 | IMT262 | .......... .......... .......... .......... ........ | | CP5 | IMT339 | .......... .......... .......... .......... ........ | | CP7 | IMT322 | .......... .......... .......... .......... ........ | | HP2 | IMT144 | .......... .......... .......... .......... ........ | | HP6 | IMT224 | .......... .......... .......... .......... ........ | | HP8 | IMT225 | .......... .......... .......... .......... ........ | | HP9 | IMT226 | .......... .......... .......... .......... ........ | | CB1 | RR066 | .......... .......... .......... .......... ........ | | CB2 | RR065 | .......... .......... .......... .......... ........ | | CB4 | RR061 | .......... .......... .......... .......... ........ | | CB6 | BH402 | .......... .......... .......... .......... ........ | | HB2 | RR072 | .......... .......... .......... .......... ........ | | HB3 | RR055 | .......... .......... .......... .......... ........ | | HB4 | RR059 | .......... .......... .......... .......... ........ | | HB6 | RR050 | .......... .......... .......... .......... ........ | |
| --- | --- | --- | --- | --- | --- | --- | --- | --- | --- | --- | --- | --- | --- | --- | --- | --- | --- | --- | --- | --- | --- | --- | --- | --- | --- | --- | --- | --- | --- | --- | --- | --- | --- | --- | --- | --- | --- | --- | --- | --- | --- | --- | --- | --- | --- | --- | --- | --- | --- | --- | --- | --- | --- | --- | --- | --- | --- | --- | --- | --- | --- | --- | --- | --- | --- | --- | --- | --- | --- | --- | --- | --- | --- | --- | --- | --- | --- | --- | --- | --- | --- | --- | --- | --- | --- | --- | --- | --- | --- | --- | --- | --- | --- | --- | --- | --- | --- | --- | --- | --- | --- | --- | --- | --- | --- | --- | --- | --- | --- | --- | --- | --- | --- | --- | --- | --- | --- | --- | --- | --- | --- | --- | --- | --- | --- | --- | --- | --- | --- | --- | --- | --- | --- | --- | --- | --- | --- | --- | --- | --- | --- | --- | --- | --- | --- | --- | --- | --- | --- | --- | --- | --- | --- | --- | --- | --- | --- | --- | --- | --- | --- | --- | --- | --- | --- | --- | --- | --- | --- | --- | --- | --- | --- | --- | --- | --- | --- | --- | --- | --- | --- | --- | --- | --- | --- | --- | --- | --- | --- | --- | --- | --- | --- | --- | --- | --- | --- | --- | --- | --- | --- | --- | --- | --- | --- | --- | --- | --- | --- | --- | --- | --- | --- | --- | --- | --- | --- | --- | --- | --- | --- | --- | --- | --- | --- | --- | --- | --- | --- | --- | --- | --- | --- | --- | --- | --- | --- | --- | --- | --- | --- | --- | --- | --- | --- | --- | --- | --- | --- | --- | --- | --- | --- | --- | --- | --- | --- | --- | --- | --- | --- | --- | --- | --- | --- | --- | --- | --- | --- | --- |

**Figure S1:** Alignment of the 447 bp *L. infantum* (*=L. chagasi*) kDNA minicircle sequences obtained for two reference strains, PPT5 and IPT1, and ten *L. infantum* strains, isolated from human and canine hosts from Brazil and Portugal. Bases labeled in gray indicate polymorphisms found only in the sequence of Reference strains. Bases marked in yellow indicate polymorphisms related with the strains’ geographic origin, while bases marked in red indicate polymorphic sites related to both, the geographic and reservoir origin of the analyzed strains. The strains codes are indicated on the left.
