# Supplementary material for: KDNA Genetic Signatures Obtained by LSSP-PCR Analysis of Leishmania (Leishmania) infantum Isolated from the New and the Old World
Source: PLoS One. 2012 Aug 17;7(8):e43363. doi: 10.1371/journal.pone.0043363 (PMC3422226; doi:10.1371/journal.pone.0043363)
Supplement: Table S1 — Relative size in base pairs of main DNA fragments of the 447 bp kDNA minicircle fragment LSSP-PCR genotypes from Leishmania infantum strains using MC1 primer. The bands shared among the kDNA signatures are marked in gray. (DOC) [file pone.0043363.s002.doc]

**Table S1**: Relative size in base pairs of main DNA fragments of the 447 bp kDNA minicircle fragment LSSP-PCR genotypes from *Leishmania infantum* strains using MC1 primer. The bands shared among the kDNA signatures are marked in gray.

| KDNA Genotypes | Strains isolated from human reservoirs | DNA fragments size | Strains isolated from canine reservoirs | DNA fragments size |
| --- | --- | --- | --- | --- |
| I | HB1, HB2, HB5, HB9, HP2, HP7 | 1619, 564, 510, 484, 420, 376, 341, 307, 281, 264, 242, 226, 207, 168, 154, 141, 126, 108, 93 | CB1, CB2, CB8, CB9, CP1, CP3, CP10 | 1435, 1009, 552, 490, 441, 380, 311, 285, 269, 171, 142 108 |
| II | HB7, HP1, HP10 | 1619, 564, 510, 477, 420, 336, 291, 264, 242, 226, 175, 154, 141, 132, 126, 108 | CP5, CP7 | 1435, 1009, 552, 490, 441, 434, 363, 308, 301, 266, 232, 186, 171, 142 108 |
| III | HB3, HB6, HB10, HP6 | 1619, 564, 510, 484, 436, 390, 361, 322, 287, 272, 242, 226, 188, 154, 141, 132, 108, 93 | CB7, CP2, CP4, CP8 | 1435, 1009, 552, 490, 441, 380, 337, 321, 311, 283, 269, 236, 196, 171, 142, 135, 108 |
| IV | HB4, HP9 | 1619, 564, 510, 484, 465, 390, 341, 306, 287, 264, 242, 226, 188, 154, 141, 126, 114, 93 | CB5, CB6, CP6, | 1435, 1009, 552, 490, 413, 380, 361, 327, 293, 273, 258, 190, 171, 142 108 |
| V | HP4, PP75 | 1619, 564, 510, 484, 446, 420, 390, 361, 339, 287, 252, 226, 220, 188, 159, 141, 93 | CB3, CP9 | 1435, 1009, 552, 490, 441, 370, 356, 334, 277, 254, 232, 218, 186, 171, 142 108 |
| VI | HP5 | 1619, 564, 510, 484, 446, 420, 390, 363, 322, 306, 281, 264, 237, 194, 175, 154, 141, 135, 122, 108, 93 | CB10 | 1033, 434, 407, 373, 356, 301, 293, 266, 255, 225, 186, 171, 135, 142, 135, 108 |
| VII | HB8 | 1619, 564, 510, 484, 420, 376, 339, 287, 274, 264, 242, 226, 188, 154, 141, 93 | CB4 | 1197, 946, 525, 457, 422, 391, 359, 301, 258, 236, 171, 155, 142, 135, 108 |
| VIII | HP3 | 1619, 564, 510, 484, 363, 337, 307, 264, 242, 220, 188, 168, 141, 93 | IPT1 | 1327, 674, 514, 490, 422, 337, 323, 301, 285, 269, 236, 196, 186, 155, 142, 135, 108 |
| IX | HP8 | 1619, 564, 510, 484, 390, 363, 322, 281, 264, 226, 201, 181, 172, 141, 126, 93 |  |  |
